# Trends in Precision Medicine and Pharmacogenetics as an Adjuvant in Establishing a Correct Immunosuppressive Therapy for Kidney Transplant: An Up-to-Date Historical Overview

**DOI:** 10.3390/ijms26051960

**Published:** 2025-02-24

**Authors:** Riccardo Belardi, Francesca Pacifici, Matteo Baldetti, Silvia Velocci, Marilena Minieri, Massimo Pieri, Elena Campione, David Della-Morte, Giuseppe Tisone, Alessandro Anselmo, Giuseppe Novelli, Sergio Bernardini, Alessandro Terrinoni

**Affiliations:** 1 Department of Experimental Medicine, University of Rome Tor Vergata, Via Montpellier 1, 00133 Rome, Italy; belardiriccardo92@gmail.com (R.B.); matteobaldetti@gmail.com (M.B.); velocci.silvia@gmail.com (S.V.); marilenami@gmail.com (M.M.); massimo.pieri@uniroma2.it (M.P.); bernards@uniroma2.it (S.B.); 2 Department of Human Sciences and Quality of Life Promotion, San Raffaele University, 00166 Rome, Italy; pacifici.francesca@gmail.com (F.P.); david.dellamorte@uniroma2.it (D.D.-M.); 3 Interdisciplinary Center for Advanced Studies on Lab-on-Chip and Organ-on-Chip Applications (ICLOC), University of Rome Tor Vergata, Via Montpellier 1, 00133 Rome, Italy; 4 Dermatology Unit, Policlinico Tor Vergata, System Medicine Department, University of Rome Tor Vergata, Via Montpellier 1, 00133 Rome, Italy; elena.campione@uniroma2.it; 5 Department of Neurology, Evelyn F. McKnight Brain Institute, Miller School of Medicine, University of Miami, Miami, FL 33136, USA; 6 Department of Biomedicine and Prevention, University of Rome Tor Vergata, Via Montpellier 1, 00133 Rome, Italy; novelli@uniroma2.it; 7 Department of Surgery, University of Rome Tor Vergata, Via Montpellier 1, 00133 Rome, Italy; tisone@med.uniroma2.it (G.T.);

**Keywords:** kidney transplant, immunosuppressive therapy, pharmacogenetic

## Abstract

Kidney transplantation is currently the treatment of choice for patients with end-stage kidney diseases. Although significant advancements in kidney transplantation have been achieved over the past decades, the host’s immune response remains the primary challenge, often leading to potential graft rejection. Effective management of the immune response is essential to ensure the long-term success of kidney transplantation. To address this issue, immunosuppressives have been developed and are now fully integrated into the clinical management of transplant recipients. However, the considerable inter- and intra-patient variability in pharmacokinetics (PK) and pharmacodynamics (PD) of these drugs represents the primary cause of graft rejection. This variability is primarily attributed to the polymorphic nature (genetic heterogeneity) of genes encoding xenobiotic-metabolizing enzymes, transport proteins, and, in some cases, drug targets. These genetic differences can influence drug metabolism and distribution, leading to either toxicity or reduced efficacy. The main objective of the present review is to report an historical overview of the pharmacogenetics of immunosuppressants, shedding light on the most recent findings and also suggesting how relevant is the research and investment in developing validated NGS-based commercial panels for pharmacogenetic profiling in kidney transplant recipients. These advancements will enable the implementation of precision medicine, optimizing immunosuppressive therapies to improve graft survival and kidney transplanted patient outcomes.

## 1. Introduction

The allogenic transplantation of solid organs is a standard procedure for patients with end-stage organ diseases. Although the transplanted organ compensates for deficient body functions, its allogenic nature can provoke an immune response, potentially leading to rejection. Managing the immune response is fundamental to achieving long-term success in organ transplantation.

### 1.1. Immunity and Transplantation

As demonstrated by numerous studies, allograft rejection is a complex process requiring the interplay of both innate and adaptative components of the immune response [1,2]. The key immunological players involved in and the mechanisms underlying graft rejection are rooted in two systems: the primitive defense mechanisms grouped under innate (or natural) immunity, and adaptive immunity.

The innate immunity system represents the first line of defense against microbes, acting rapidly to eliminate pathogens in a non-specific manner [3]. It consists of complement system proteins, which act as physical and chemical barriers, and immune cells, including macrophages, neutrophils, natural killer cells, and innate lymphoid cells. Cells of the innate immune response express a limited set of receptors known as the pattern-recognition receptors. These receptors recognize pathogen-associated molecular patterns during infection [4,5]. It has been demonstrated that cell injury can release damage-associated molecular patterns, which includes several cellular and nuclear proteins. This release triggers the activation of innate immune cells. Cell injury commonly occurs in transplanted solid organs, after surgery and during the early stages of graft rejection [4,5,6], leading, therefore, to the sensitization and activation of the innate immune system.

This activation further stimulates adaptative immunity, which serves as the primary effector in the rejection process. Adaptative immunity involves B-cell and T-cell compartments, and is characterized by antibody secretion. Specific immune cells, including lymphocytes, mediate the stated activity. Remarkably, adaptive immunity shows a wide variety of receptors in both T- and B-cells. This diversity arises from the somatic recombination of gene segments, enabling the immune system to react very specifically to a vast array of potential antigens [7]. Moreover, the effector cells of adaptative immunity can retain the memory of antigens, reacting more strongly and rapidly to a second or repeated exposure to the same antigen. While this memory response is very helpful against repeated infections, it represents a significant risk in organ transplants [1,6]. Host-specific receptors recognize graft cells as foreign, leading to their attack and destruction, ultimately resulting in organ rejection [8].

### 1.2. Kidney Transplantation and Immunosuppression

Over the past two decades, the life expectancy of subjects undergoing kidney transplantation has considerably lengthened, also thanks to the development and use of new immunosuppressive drugs. These medications suppress the immune response to the transplanted organ, which, in the absence of such intervention, would be recognized as “foreign” and subsequently rejected.

Advances in kidney transplantation have been achieved over the years, despite limited immunosuppression options in earlier periods. The scientific literature outlines the evolution of kidney transplantation alongside advancements in immunosuppressive therapy through four distinct stages [9].

1. The early period (1950s): this stage focused mainly on the immunological matching between host and donor rather than effective immunosuppression. Many early transplants occurred between human leukocyte antigen (HLA)-identical siblings, with no immunosuppression requirement, or between genetically close donor–recipient pairs. Treatment, during this time, involved corticosteroids, 6-mercaptopurine, and radiation therapy [10]. However, the survival rates were very low.
2. The Azathioprine (AZA) era (1962–1980): the introduction of AZA extended graft survival to approximatively 60% [11].
3. The Cyclosporine (CsA) era (1980s): the adoption of this immunosuppressive drug, a calcineurin inhibitor (CNI), lead to a significant reduction in the rejection rates below 50%, and to an increased graft survival over 85% [12].
4. The contemporary period: the immunosuppressive therapy has been further improved with the approval drugs such as Tacrolimus (TAC), Mycophenolate mofetil, rabbit antithymocyte globulin, and IL-2 receptor blockers, Basiliximab and Daclizumab. Combinations of these therapies improved the one-year rejection rate to 10–15% and survival graft rates to 95% [13]. While newer therapeutic agents and antibodies have been developed, their widespread use remains limited due to concerns about their efficacy compared to existing treatments.

Over the years, immunosuppressive therapies have focused on preserving the function of transplanted organs and preventing rejection by modulating the immune system. The primary approaches employed are summarized below:

- Reduction in lymphocytes, by using anti-lymphocyte sera;
- Receptor inhibition with monoclonal antibodies (Basiliximab and Daclizumab), with consequent reduction in lymphocyte proliferation;
- Use of potent anti-inflammatory drugs, such as corticosteroids;
- Use of anti-proliferative drugs, such as Mycophenolate, which inhibit the synthesis of purine bases, blocking DNA synthesis and cell duplication;
- CNI, leading to the blockage of lymphocyte activation (CsA and TAC);
- Inhibition of the “mammalian target of rapamycin” (m-Tor), a protein that regulates the growth, proliferation, motility, and survival of immune cells.

## 2. Trends in Precision Medicine

Precision medicine (PM), an approach leveraging an individual’s genetic makeup, surroundings, and lifestyle for optimal disease prevention or treatment, is complemented by the older term “personalized medicine,” aiming to tailor strategies to an individual’s disease processes or symptoms, emerging as a critique of reductionist medical practices [14,15]. The shift from a one-size-fits-all treatment framework, driven by PM, sparks a reasonable desire for a more accurate diagnosis and personalized care, historically aligning with infectious disease management goals, emphasizing causative organism identification and data repositories for specific treatments, integrating technology over time [16,17]. Sir William Osler’s century-old goals of medicine, seeking to unveil secrets, trace disease origins, and correlate vast knowledge for swift application, find resonance in the accumulation of substantial health data and advancements in DNA sequencing, decreasing costs and fostering innovation for individualized healthcare delivery [18]. PM integrates advanced technology, creating a data ecosystem, combining clinical phenotypes and biological information to develop a new molecular biology-based disease taxonomy. The 2011 National Research Council Report underscores PM’s potential impact on disease awareness, accurate diagnoses, refined treatments, and innovative therapies [14]. PM extends to non-communicable diseases, refining disease taxonomy for specific pathogenesis understanding in conditions like heart disease, cancer, and obesity, opening avenues for maximizing treatment efficacy while minimizing adverse events [19].

Innovative trial designs, such as basket or umbrella trials, streamline drug discovery in PM studies, raising concerns within the public health community about applicability and credibility, particularly regarding genomic emphasis and potential limitations [20,21,22,23,24].

Delving into PM’s potential role in diagnosis and treatment is crucial for a comprehensive assessment of its impact on population health. The advances in molecular medicine advocate for a new disease taxonomy based on molecular biology, shaping the future of PM [25]. PM aims to identify unique, safe, and effective treatments based on genetics and environment [26,27]. The Kidney PM Project targets chronic kidney disease and acute kidney injury [28]. While genomic and molecular profiling in transplantation offer unprecedented opportunities, the realistic approach of PM necessitates a careful assessment of potential implications to avoid restrictions or discrimination against otherwise healthy individuals [29].

### Applications of Precision Medicine in Kidney Transplants

Kidney transplantation stands as the preferred treatment for patients with end-stage renal disease (ESRD), offering a lifeline to those on the waiting list in the United States [30]. However, despite approximately 140,165 patients awaiting kidney transplants, in the United States, only 26,309 were performed in 2022. Although the number of deceased donor kidney transplants increased, the COVID-19 pandemic slowed down the procedure [31]. While mortality for ESRD patients post-transplant is lower than those on maintenance dialysis, it remains about fourteen times higher in the first year and four times higher thereafter compared to the general population [32,33]. The challenges extend to long-term outcomes, influenced by factors such as donor characteristics, allograft quality, and recipient-specific variables, including immunosuppressive response and the development of donor-specific antibodies (DSAs) [34,35,36]. Immunomodulatory drugs, with their narrow therapeutic index and variable PK, require personalized regimens [37].

One of the key areas of PM in transplantation is pharmacogenetic testing, which has been explored to optimize immunosuppressive therapy dosing, particularly for TAC [38]. In general, genotype-guided dosing can help achieve target drug concentrations more efficiently in transplant recipients; for example, it can help with accounting for the influence of *CYP3A5* polymorphisms on TAC metabolism [39]. However, further randomized trials are needed to confirm whether this approach translates into improved clinical outcomes in terms of rejection rates and drug-related toxicities [40]. Beyond pharmacogenetics, biomarker-driven immunosuppression strategies are emerging as promising tools to predict the risk of rejection and to optimize immunosuppressive regimens [40]. Molecular biomarkers such as donor-derived cell-free DNA (dd-cfDNA) [41,42,43] and urinary chemokines (CXCL-9, CXCL-10) [44] provide non-invasive monitoring for allograft health and subclinical rejection, potentially reducing reliance on protocol biopsies. Additionally, predictive modeling based on HLA DSAs and antibody-verified eplet mismatches, coupled with machine learning approaches, is being investigated to stratify rejection risk and develop individualized immunosuppressive protocols [45,46].

A major challenge in kidney transplantation remains medication non-adherence, a leading cause of late allograft loss. Studies indicate that targeted interventions based on patient-specific behavioral patterns and risk stratification significantly improve adherence to immunosuppressive therapy and long-term graft function [47]. Personalized approaches, incorporating digital adherence monitoring tools and patient education programs, are being integrated into clinical practice to mitigate non-adherence and enhance outcomes. In this context, next-generation sequencing (NGS) has emerged as a pivotal tool in transplant medicine, enabling high-throughput immune repertoire sequencing to characterize alloreactive T-cell receptor (TCR) diversity and clonal expansion during rejection episodes [48,49,50]. By tracking TCR clonotypes over time, NGS provides a more precise method for immune surveillance, complementing traditional biopsy-based assessments [51,52]. Additionally, single-cell RNA sequencing has enhanced our understanding of intragraft immune responses, offering the potential for early detection of rejection and immune tolerance mechanisms [53]. The integration of these genomic technologies into routine transplant monitoring may refine risk stratification and facilitate preemptive intervention before irreversible graft injury occurs [54].

Artificial intelligence (AI) applications are further advancing transplant PM by integrating multi-omics data with clinical variables to improve predictive modeling [55]. Machine learning algorithms have been developed to analyze patterns of rejection based on histopathological and molecular biomarkers, increasing diagnostic accuracy and reducing interobserver variability [56].

AI-driven tools are also being explored to optimize immunosuppressive therapy, tailoring regimens to individual patients based on real-time immunological profiling [57]. Additionally, deep learning models applied to the histopathological images of allograft biopsies have demonstrated high accuracy in identifying rejection phenotypes, potentially reducing interobserver variability and improving diagnostic precision [58]. These advancements highlight the growing role of PM in improving kidney transplant outcomes. By integrating pharmacogenomics, biomarker-based monitoring, artificial intelligence, and patient-centered adherence strategies, transplantation is shifting towards a more personalized approach. This paradigm not only reduces rejection episodes but also enhances patient stratification and optimizes long-term immunosuppressive therapy, ultimately improving allograft survival and quality of life for recipients.

Infections and cardiovascular issues further contribute to suboptimal outcomes, emphasizing the need for tailored immunosuppression and antimicrobial strategies [59,60,61,62,63]. Additionally, the integration of cardiovascular medications guided by actionable genetic information supports the concept of precision prescribing in kidney transplant recipients, promising improved efficacy and reduced drug interactions [64].

In the realm of solid organ transplantation, recipients undergo induction immunosuppressive therapy at surgery, with subsequent maintenance therapy. The purpose is to dampen allogeneic responses, involving glucocorticoids, T-cell depletion, and B-cell or plasma-cell depletion based on perceived rejection risk [65]. Despite the evolution in available induction agents, defining the most efficacious regimens lacks evidence from head-to-head randomized controlled trials. Prescribing patterns follow guidelines like the 2009 Kidney Disease Improving Global Outcomes, providing moderate evidence strength [66]. TAC, a CNI, serves as the backbone for maintenance therapy, showing superiority over other agents in prospective and randomized studies [67]. Yet, the side-effect profiles of maintenance immunosuppressants continue to influence the diverse transplant population. Therefore, understanding and leveraging the pharmacodynamics (PD) of these agents are pivotal for achieving more favorable clinical outcomes.

## 3. Drug Monitoring of Immunosuppression

All drugs with immunosuppressive capacity have a specific and generally not wide therapeutic range, requiring caution in their use [68]. The therapeutic range can be defined as the concentration range of the drug within which it is effective without being toxic. An effective therapy, in fact, must avoid the serious side effects associated with excessive exposure to the drug and, at the same time, it must reduce the possible rejection due to inadequate immunosuppression. For this reason, and to improve the clinical response in post-transplantation, the periodic measurement of drug concentrations in a patient’s blood has been consolidated over time. This result has been achieved monitoring the serum concentration of administrated immunosuppressive drugs (Therapeutic Drug Monitoring, TDM), which is generally carried out in clinical chemistry laboratories.

In the late 1990s, measurement techniques based on chromatographic separation were introduced to improve sensitivity and analytical specificity [69]. Chromatographic separation (or, in short, chromatography) allows for separating the substances contained within even a complex mixture and, in the biochemical-clinical field, allows for separating and analyzing drugs and substances contained within a blood sample.

The liquid chromatography associated to mass spectrometry methods (LC-MS) allow for obtaining comparable results among centers that carry out the measurement of the drug with the same technology, facilitating the comparability and standardization of the results and, consequently, the exchange of relevant information for the management of the patient and for the improvement of the dosage regimen [70,71]. A personalized dosage leads to a reduction in the side effects due to an excess of the drug, and to a reduction in the risk of organ rejection due to an insufficient quantity of the drug. Finally, the improved analytical performance, which minimizes the interference caused by cross-reactivity with endogenous and exogenous substances, reduces the need for repeated sampling and therapeutic confirmation measurements. In TDM, the methods and timing with which the patient’s blood sample is taken are particularly important. After a patient takes a drug, the drug’s concentration in the blood increases reaching a maximum peak, and then progressively decreases and reaches the minimum concentration level (basal level), which must be maintained until the next dose [71].

With regards to the immunosuppressants described in this paper, drug concentrations can be evaluated using immunoassays or liquid chromatography coupled with mass spectrometry (LC-MS/MS) [70,72,73]. For the drugs TAC (FK506), Sirolimus (SRL) (rapamune), and Everolimus (EVR), a single basal blood draw is sufficient [74]. However, for the monitoring of CsA and mycophenolic acid (MPA) [75], two samples are required for the former (basal and two hours post-administration), and three or four samples for the latter, respectively.

For the quantification of EVR levels in patients’ blood, LC-MS/MS is the preferred method. Nevertheless, immunoassays such as FPIA (Fluorescence Polarization Immunoassay) and CMIA (Chemiluminescent Microparticle Immunoassay) are still utilized in transplant centers that lack access to chromatographic tests. One drawback of immunoassays for measuring immunosuppressant blood concentrations is their positive bias compared to chromatographic techniques. This bias is mainly caused by cross-reactivity with metabolites of the target molecule. Specifically, the QMS (Quantitative Microsphere System) EVR immunoassay shows cross-reactivity of 59–63% with 40-phosphatidylcholine-EVR and <20% with five other metabolites, leading to an overestimation of drug concentrations in patient samples. Additionally, the structural similarity between SRL and EVR can result in cross-reactivity and biased measurements when switching therapies [72]. Most transplant centers use both LC-MS/MS and immunoassays for the measurement of SRL concentrations in whole blood, although High-Performance Liquid Chromatography (HPLC) is also employed. HPLC enables measurement of the parent drug but is time-consuming [76]. Aside from LC-MS/MS, TAC can be measured using various immunoassays, among which CMIA has become the preferred choice due to its low bias compared to chromatography, its improved precision, and its lack of interference from factors like hematocrit or bilirubin [70]. Scientific literature highlights differences in the preferred sample matrix and the analytical approaches for monitoring other immunosuppressants, including MPA. Since MPA is primarily distributed in the extracellular space, serum or plasma is the appropriate sample matrix for measurement. MPA levels can be determined using HPLC, UHPLC (Ultra-High-Performance Liquid Chromatography), or the IMPDH inhibition assay. The latter leverages the in vivo mechanism of action, providing higher analytical specificity than immunoassays [73].

CNS was the first immunosuppressant to be measured in transplantation [77]. While LC-MS remains the gold-standard method for its measurement due to its high sensitivity and selectivity, most TDM laboratories rely on immunoassays. Given that multiple venous blood collections are required, alternative methods such as dried blood spots (DBS) or volumetric absorptive microsampling (VAMS) can be viable options. Microsampling is particularly advantageous for pediatric patients, as it reduces the burden of blood collection [78].

The first method to measure AZA’s two primary metabolites (6-TGN and 6-MMPR) was developed by Lennard and Singleton, later simplified by Dervieux and Bolieu. This technique is based on HPLC and is commonly used for TDM of AZA [79].

In conclusion, the choice of HPLC-MS/MS and UHPLC-MS/MS technologies represents an undoubted advantage for patients who can be guaranteed by an accurate, sensitive, and specific evaluation of immunosuppressive drugs. These technologies, even if the initial investment can be considerable, allow for obtaining an appreciable economic saving while maintaining and improving the service to the patient [74,80]. Future studies and method validations are necessary to further expand the range of accessible and cost-effective TDM approaches, alleviating economic and patient burdens in transplantation medicine.

## 4. Pharmacogenetics of Kidney Transplantation

Inter- and intra-individual variability, arising from several factors, can be detected in transplant patients treated with immunosuppressants. These factors influence drug metabolism and transport and include age, hematocrit levels, hepatic and renal function, interaction with other drugs, and genetic polymorphisms, among others [81,82]. In particular, genetic factors have been highlighted as significant contributors to differences in drug metabolism and transplant outcomes across individuals. Currently, the wide variability in PK and PD represents a critical challenge. Genetic heterogeneity, by altering these parameters, could potentially lead to significant toxicity or reduced therapeutic efficacy. The most commonly used immunosuppressants in organ transplant include CsA, MPA, TAC, EVR, SRL, and AZA [83,84,85].

As previously stated, the effectiveness of immunosuppressive therapy in preserving graft function depends on maintaining drug levels within a therapeutic range. A patient’s metabolism influences dosage and metabolite concentrations. Recent studies have examined the relationship between genetic factors, drug PK, and therapy outcomes. These studies suggested that genetically determined polymorphisms in xenobiotic-metabolizing enzymes, transport proteins, and in some cases, drug targets partially explain inter-individual variability [86,87].

### 4.1. TAC

**Figure 1:**
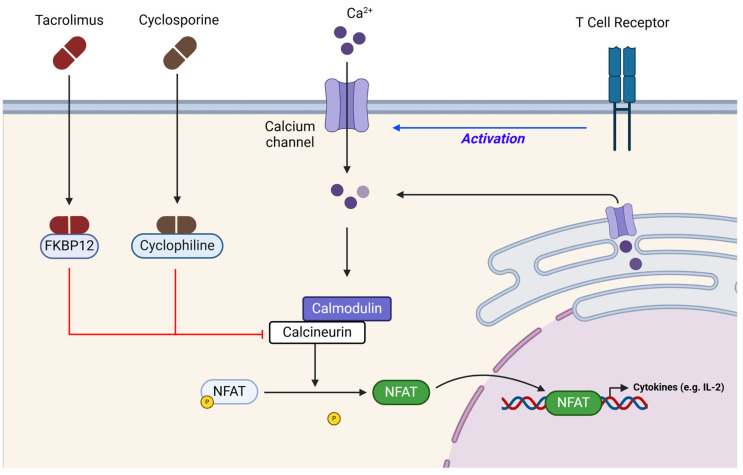
Immunosuppressive function of tacrolimus (TAC) and cyclosporine (CsA), focused on T-cell activation. Both immunosuppressive drugs inhibited the phosphatase calcineurin, thus avoiding the dephosphorylation of NFAT and blunting the transcription of IL-2. FKBP12: 12-kDa FK506-binding protein; IL-2, interleukin-2; NFAT, nuclear factor of activated T-cells. Created with BioRender.com (accessed on 7 January 2025).

TAC is now a drug considered the first-line medication against immune response in kidney transplants. In particular, it reduces several target genes’ expression (such as Interleukin-2) and T-cell proliferation, indirectly leading to the inhibition of B-cell-mediated antibody release [88] (Figure 1).

The narrow therapeutic window of TAC is influenced, at least in part, by its metabolism mediated by the Cytochrome P450 Family 3 Subfamily A (CYP3A), composed of four relevant genes: *CYP3A4*, *CYP3A5*, *CYP3A7*, and *CYP3A43* [89]. Several single-nucleotide polymorphisms (SNPs) related to *CYP3A* have been identified, revealing the impact of these genetic variants in TAC PK. In particular, the most relevant findings are related to the CYP3A5. Interestingly, the *rs776746* SNP (A>G; loss of function) leads to individual classification as an “expresser” (whether at least one wild-type *1 allele is present) called also CYP3A5*1/*1 (AA) and CYP3A5*1/*3 (AG), with normal TAC metabolism, or as a “nonexpresser” (when only the mutant *3 allele exists), known as CYP3A5*3/*3 (GG), with reduced TAC metabolism, due to the loss of functional CYP3A5 protein [90]. Accordingly, previous studies conducted on Asian populations demonstrated that CYP3A5 expressers required greater TAC dosages to reach the target concentration, compared to non-expressers [91,92]. Similar results were also obtained by Ferraris and colleagues, in 48 pediatric patients subjected to kidney transplant [93]. The authors found a more than two-fold higher TAC requirement in CYP3A5*1/*1 and *1/*3 carriers compared to CYP3A5*3/*3, with increased blood TAC levels in the latter (with enhanced concentration/dose, Co/D, ratio).

Recently, Chauhan and colleagues, published a meta-analysis including 18 studies correlating the genetic variation of *CYP3A5* in TAC PK [89]. The main finding was the ethnicity difference in the presence of *CYP3A5* genetic variants among Asian and Caucasian populations. In particular, the expressers were 63.94% for Asians and 81.44% for Caucasians, highlighting the relevance of ethnicity in allograft transplantation outcomes. Moreover, the authors interestingly suggested considering the presence of *CYP3A5* polymorphism for TAC dosage rather than body weight to reach the therapeutic concentration of TAC, reducing the incidence of transplant rejection and drug side effects.

However, other genes are involved in TAC metabolism and should potentially be considered to better personalize TAC therapy in transplanted patients. Among these, *CYP3A4* is a gene highly expressed in both hepatic and small intestinal tissues. Research by Tamashiro et al. indicated that the CYP3A4*1G (*rs2242480*) polymorphism significantly influences TAC concentrations, suggesting its potential role in modulating the drug’s pharmacokinetics (PK) [94]. Furthermore, the *CYP3A4 rs4646437* variant has been implicated in the metabolism of multiple drugs, including TAC [95]. A recent study by Dong et al. demonstrated that the *CYP3A4 rs4646437* and *rs2242480* SNPs in transplant recipients (intestinal expression) are significantly associated with TAC concentrations during the early post-transplant phase [96]. Two studies investigating the role of genetic polymorphisms in TAC metabolism among Chinese renal transplant patients identified a significant impact of the *CYP3A4 rs4646437* and CYP3A4*1G (*rs2242480*) polymorphisms on TAC concentrations post-transplantation [95,97]. Combined with the findings from Dong et al., these studies suggest that *CYP3A4 rs4646437* and CYP3A4*1G (*rs2242480*) polymorphisms in transplant recipients (intestinal expression) could serve as crucial biomarkers for TAC concentration variability during the first month following liver transplantation. The *CYP3A4* gene also has two relevant polymorphisms associated with opposite functions. Patients carrying the CYP3A4*1B (*rs2740574*) allele exhibit increased TAC metabolism, requiring a higher TAC dosage. Conversely, carriers of the CYP3A4*22 (*rs35599367,* C>T) allele required a lower TAC dosage [98,99]. The latter has been reported to significantly affect CYP3A4 expression, suggesting its potential role as a biomarker for predicting the response to CYP3A4-metabolized drugs [100]. The impact of CYP3A4*22 on TAC PK has been extensively studied [101,102,103]. However, its prevalence in East Asian populations is particularly low (https://www.internationalgenome.org/ (accessed on 10 February 2025)).

Other genes that may influence TAC efficiency include *CYP3A7* (*rs10211* and *rs2257401*). In depth, recent findings indicate that kidney transplant recipients carrying the *CYP3A7 rs10211 AA* genotype exhibit nearly double the TAC concentration compared to non-carriers, suggesting a potential need for lower TAC dosages [96]. These results align with previous studies [104,105]. Additionally, the *CYP3A7 rs10211* polymorphism has been associated with TAC PK in pediatric patients with nephrotic-range proteinuria [105]. Collectively, these studies suggest that *CYP3A7 rs10211* may serve as a biomarker for TAC blood level variability. Regarding the *rs2257401* polymorphism, its expression results in a missense mutation located in the third exon of the *CYP3A7* gene, where the substitution of cytosine (C) with guanine (G) leads to an amino acid change from threonine (Thr) to arginine (Arg) in the translated protein. Previous studies have associated the *CYP3A7 rs2257401* polymorphism with TAC concentration variability in adult kidney transplant recipients [106]. However, the most recent study by Dong et al. indicates that the *CYP3A7 rs2257401* polymorphism does not significantly affect TAC concentrations during the early post-transplant period [96].

Another recently studied gene is *ABCB1* (also known as *MDR1*), located at 7q21.12, encoding P-glycoprotein, a transmembrane transporter involved in drug distribution and efficacy [107]. Polymorphisms such as *ABCB1 3435C>T*, *ABCB1 1236C>T*, and *ABCB1 2677G>T/A* influence TAC bioavailability, with ethnic variability affecting allele distribution and drug response [108,109]. According to the study by Rotarescu et al. [110], in Romanian kidney transplant recipients, the C allele frequencies for *ABCB1 C1236T* and *C3435T* (70.4% and 75.3%) differed from those observed in other populations, highlighting genetic diversity [111].

According to the previously mentioned study, patients with the 3435 TT genotype required lower TAC doses in the first six months post-transplant, whereas those with the 3435 CC genotype exhibited lower dose-adjusted TAC concentrations in the subsequent six months [112,113]. However, some studies found no significant association between *ABCB1* polymorphisms and TAC trough levels, suggesting a complex interaction between genetic and environmental factors [114,115]. Over time, the genetic influence on TAC metabolism declines, while non-genetic factors, such as age and clinical status, become more prominent. The genetic analysis of *ABCB1* polymorphisms could enhance personalized TAC dosing, reducing toxicity and rejection risks, but further research with larger, controlled studies is needed to refine pharmacogenetic models [116,117].

Furthermore, the polymorphisms *rs181781* of IL-3 and *rs4553808* on Cytotoxic T-Lymphocyte Antigen 4 have been associated with reduced TAC metabolism, necessitating lower drug dosages. Additionally, carriers of the Cytochrome P450 Oxidoreductase haplotype *rs1057868-rs2868177* (GC-GT) show increased blood TAC levels [118,119].

The previous findings are mainly based in Caucasian and Asian subjects. It should also be mentioned that the Black population exhibited a higher TAC clearance compared to the white population [120], suggesting that higher dosages should be administered compared to other populations.

In conclusion, to better personalize immunosuppressive therapy in kidney-transplanted patients receiving TAC, the analysis of the CYP3A5*3 phenotype should be considered.

### 4.2. CsA

CsA was first identified in the 1970s as a cyclic undecapeptide metabolite produced by the fungus *Tolypocladium inflatum* [121,122]. Among its analogs, CsA, a lipophilic cyclic peptide composed of 11 amino acids, became the first CNI approved by the FDA in 1983 for immunosuppression in kidney transplantation [123]. CsA significantly improved transplant outcomes by reducing acute rejection rates and increasing one-year post-transplant survival to approximately 90% [35,124] (Figure 1), marking a pivotal advancement in transplantation medicine.

CsA is absorbed by intestinal epithelial cells, although some is actively effluxed back into the lumen by membrane-bound P-glycoprotein 1. It undergoes extensive hepatic metabolism, predominantly via the cytochrome P450 enzymes CYP3A4 and CYP3A5, yielding over 30 metabolites, 90% of which are excreted via bile [123]. The PK of CsA exhibit significant interindividual variability due to variations in CYP3A enzyme activity, which can differ up to 20-fold among individuals [125,126]. Genetic polymorphisms such as *CYP3A422* and *CYP3A5*3 have been linked to altered CsA clearance [127,128], though these variants explain only a portion of the variability, with transcriptional regulators like HNF3γ (Hepatocyte nuclear factor 3γ) and PXR (pregnane X receptor) playing additional roles [129,130,131].

Recent studies have examined other genetic factors affecting CsA metabolism and transport. For instance, Zhai et al. [132] investigated the *TSPYL1* (Testis-specific Y-encoded-like protein 1) variant *rs3828743* and observed reduced CYP3A4 activity in kidney transplant patients, contrasting with in vitro findings that associated the variant with increased CYP3A4 expression [132]. These discrepancies may arise from tissue-specific differences in CYP3A4 and TSPYL1 expression, as both are highly expressed in the liver, the site of CsA metabolism, but minimally expressed in the prostate, the tissue used in prior in vitro research [133]. Additionally, regulators such as REST and ZBTB7A influence TSPYL and CYP3A4 activity, with emerging evidence suggesting a role for TSPYL4, especially when TSPYL1 function is impaired [133,134]. However, studies to date, including those using CYP3A5*3 as a marker, have not identified significant associations between *TSPYL1 rs3828743* and CYP3A4 activity, highlighting the complexity of CsA pharmacogenetics.

The role of ABCB1 and CYP3A5 in CsA PK has also been widely studied. Some studies suggest that the presence of CYP3A*5/*1 and/or CYP3A4*1/*B alleles is associated with higher CsA dose requirements and lower dose-normalized trough levels (C0/D ratio). Conversely, variants like CYP3A4*22 have been linked to increased CsA concentrations, suggesting that specific haplotypes could inform dosing strategies [86]. While some studies report no pharmacogenetic associations between CYP3A enzymes, ABCB1, and CsA PK measures [135,136], others advocate for preemptive genotyping as a tool for optimizing CsA therapy in transplant patients.

In association with the polymorphisms of *ABCB1*, another relevant player in CsA metabolism is the steroid and xenobiotic receptor (SXR), which is involved in the transcription of both cytochrome P450 and ABCB1 enzymes [137]. Specifically, a deletion of six base pairs (pb) in the promoter region of *SXR* (characterized as polymorphism *rs3842689*) has been reported to impact CsA metabolism during the transition from childhood to adulthood following kidney transplantation [138].

In conclusion, *SXR* and *CYP3A* polymorphisms should be evaluated prior to kidney transplant to determine the optimal therapeutic approach considering CsA administration.

### 4.3. SRL and EVR

**Figure 2:**
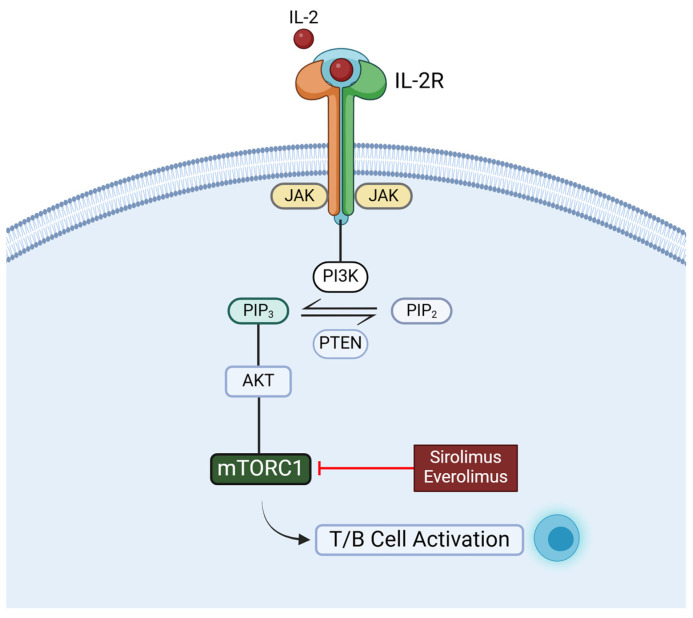
Immunosuppressive function of sirolimus (SRL) and everolimus (EVR). Both drugs inhibited the IL-2-mediated signaling pathway, by modulating the activity of mTORC1, blocking the activation of both T- and B-cells. mTORC1: mammalian target of rapamycin complex 1; IL-2, interleukin-2. Created with BioRender.com (accessed on 7 January 2025).

EVR and SRL exert their immunosuppressive effects by inhibiting the IL-2 response, thereby preventing the activation of T- and B-cells. They both bind to the FK-binding protein 12 (FKBP12), inhibiting the mammalian target of rapamycin (mTOR) pathway by directly binding to the mTOR Complex 1 (mTORC1) (Figure 2) [139].

Both drugs are metabolized by CYP3A4, CYP3A5, and CYP2C8, while *ABCB1*, a gene encoding P-glycoprotein, plays a role as a cellular efflux pump. Consequently, pharmacogenetic studies have been focused on the respective genes.

Specifically, the wild-type genotype CYP3A4*1/*1 has been linked to higher post-treatment concentration–dose ratios of SRL compared to those with CYP3A4*1B; this is probably due to a higher enzymatic activity in the patients carrying the mutant alleles. A similar result was observed in patients carrying an SNP in the intron 3 of the *CYP3A5* gene, which affects the RNA splicing with the consequent production of an enzyme with a reduced activity. This results in lower SRL concentration–dose ratios in patients with CYP3A5*1 compared to those carrying the CYP3A5*3/*3 genotype [140]. Furthermore, a thorough meta-analysis performed in 2020 by Shao et al. investigated the influence of *ABCB1 C3435T*, *C1236T*, and *G2677T/A* polymorphisms on the dose-adjusted trough level (C/D) of SRL in renal transplant recipients. Even though the homozygosity for the T allele in the *C3435T* SNP was associated with lower expression levels of P-Glycoprotein in the intestine, no association was found between the C/D ratio of SRL and *ABCB1 C3435T* polymorphism. *ABCB1 C1236T* SNP was then evaluated in all patients via the homozygous model (TT vs. CC) and it was found that dose-adjusted concentrations of SRL in Caucasian CC genotype carriers are significantly higher than in TT carriers. Moreover, patients carrying the *G2677T* homozygous genotype TT would require higher doses of SRL to reach target levels compared to those with the wild genotype GG [141].

With regard to EVR, the existing literature does not offer as much evidence compared to SRL from a pharmacogenetic standpoint. Moreover, most of the studies that were performed on kidney, liver, and heart transplantation do not support the effect of *CYP3A4* or *CYP3A5* variants on the PK of EVR. This is the case of a study by Moes et al., in which it was shown that polymorphisms in genes coding for ABCB1, CYP3A5, CYP2C8, and PXR with an allele frequency >6% do not influence EVR PK in a clinically relevant manner, and are therefore not suitable to help improve the prediction of EVR exposure [142]. The evaluation of the existing literature led to the conclusion that more data are needed to further clarify the SNPs’ impact on the SRL and EVR PK.

### 4.4. MPA

**Figure 3:**
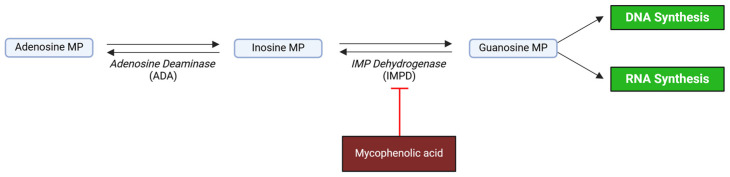
Immunosuppressive function of mycophenolic acid (MPA). It functions as an inhibitor of the enzyme inosine 5′-monophosphate dehydrogenase (IMPDH), which converts inosine 5′-monophosphate (IMP) into xanthosine 5′-monophosphate (XMP), leading to the de novo synthesis of guanine nucleotides. Created with BioRender.com (accessed on 7 January 2025).

MPA, an antiproliferative agent, is a relevant component of immunosuppressive therapy in various clinical contexts, including transplant medicine and the treatment of several autoimmune diseases [143]. MPA functions as a selective, non-competitive, and reversible inhibitor of the enzyme inosine 5′-monophosphate dehydrogenase (IMPDH), which is critical for the converting of inosine 5′-monophosphate (IMP) into xanthosine 5′-monophosphate (XMP), a key step in the de novo synthesis of guanine nucleotides [144] (Figure 3).

The cytostatic activity of MPA is particularly pronounced in lymphocytes due to their exclusive reliance on the de novo purine synthesis pathway, unlike other cells that can utilize a salvage pathway to produce guanine nucleotides [145]. This specificity makes MPA the immunosuppressant of choice in post-transplant therapeutic regimens. Over 70% of renal and cardiac transplant recipients, and more than 50% of lung transplant patients, receive MPA as part of a triple-drug immunosuppressive regimen [146].

MPA PK exhibits significant interindividual variability, primarily driven by genetic polymorphisms in the enzymes responsible for its metabolism, particularly those related to the UDP glucuronosyltransferase family, such as UGT1A9 and UGT2B7. A specific polymorphism in the *UGT1A9* gene has been linked to substantial variations in MPA PK in kidney transplant patients [147].

Peak plasma concentrations of MPA are influenced by the presence of the *UGT1A9-440C>T* allele. Furthermore, carriers of the *UGT1A9-275A* allele show pharmacokinetic differences compared to those with the T allele [148]. Both the *ABCB2* gene and the *UGT* gene family have been extensively studied for single nucleotide polymorphisms (SNPs) that affect MPA metabolism. Notably, patients with polymorphisms in *UGT1A9*, *UGT2B7*, and *MRP2* (multidrug resistance protein 2) require higher MPA doses than carriers of non-UGT SNPs [149].

Studies involving more than 300 patients demonstrated that the *UGT1A9 T-275A* polymorphism is associated with lower MPA concentrations. Conversely, analyses of 125 patients revealed that the *UGT1A9 1399 T/T* genotype correlates with higher MPA blood concentrations, enabling dose reductions.

Relevantly, when immunosuppressant therapy is prescribed, the race ethnicity of patients should be taken into account. In fact, it has been reported as although the PK of MPA is similar between Caucasians and Afro Americans, the latter need a higher MPA dosage [150]. This could be explained by the fact that Afro Americans presented a more robust immune system, but it could be also hypothesized that a different sensitivity in intracellular molecules led to MPA inhibition.

In a review of Pangmei et al., it has been reported that the Asian population reaches higher MPA levels when a similar dosage of michophenolate mofetil has been administered, suggesting that a 20–46% lower dosage of MPA, compared to that of Caucasians and Afro Americans, should be given as immunosuppressant therapy [151]. Several factors may contribute to this variability: among those, a key role is played by the different frequencies of SNPs in the enzyme involved in MPA metabolism [152]. In particular, it has been demonstrated that the polymorphism UGT1A9*1 c-440C>T/-331T>C, associated with a lower MPA clearance, was 42% in Caucasians, 8% in Africans, and 2% in Asians [120].

Based on these findings, further research is needed to clarify the impact of SNPs on MPA PK and optimize its therapeutic use [153], also considering the race and ethnicity of patients.

### 4.5. AZA

**Figure 4:**
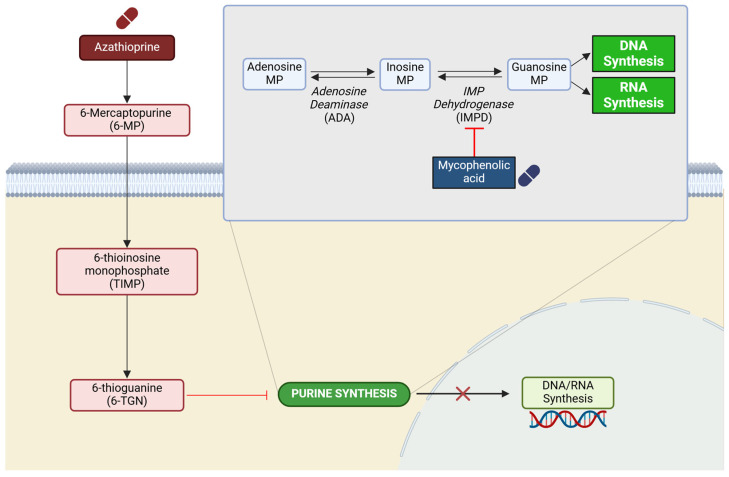
Immunosuppressive function of azathioprine (AZA). It functions by inhibiting purine synthesis. Created with BioRender.com (accessed on 7 January 2025).

AZA is an immunosuppressive agent widely used for post-transplant immunosuppression and the treatment of active rheumatoid arthritis, approved by the Food and Drug Administration (FDA) [154,155]. AZA acts as a prodrug, requiring enzymatic conversion to its active metabolites, mercaptopurine (6-MP) and thioguanine (6-TGN), which inhibit purine synthesis by incorporating it into replicating DNA, thus halting cellular division [66,156,157] (Figure 4).

The immunosuppressive and toxic effects of AZA are primarily attributed to these metabolites. AZA is rapidly absorbed through the gastrointestinal tract and metabolized in the liver, with renal excretion. This can exacerbate toxic effects in patients with impaired kidney function [155].

The metabolism of AZA is significantly influenced by two enzymes: thiopurine methyltransferase (TPMT) and nucleoside diphosphate-linked moiety X (Nudix)-type motif 15 (NUDT15). TPMT catalyzes the methylation and inactivation of 6-MP, and its activity is highly dependent on genetic polymorphisms. *TPMT* is a polymorphic gene, with over 40 known variants [158,159,160,161], and the most clinically relevant alleles include TPMT*2 (*c.238G>C*), TPMT*3A (*c.460G>A* and *c.719A>G* in *cis*), and TPMT*3C (*c.719A>G*), which account for more than 90% of cases with reduced or absent enzyme activity [162,163,164,165,166]. Approximately 10% of individuals of white ethnicity carry at least one slow metabolizer variant, resulting in toxic metabolite accumulation and an increased risk of severe myelosuppression [162]. About one in three hundred individuals are homozygous for inactivating variants, leading to complete enzyme deficiency and a high risk of life-threatening toxicity [164,165]. Heterozygous individuals also face elevated risks of bone marrow suppression and require dose adjustments guided by enzyme activity or genetic testing [160,164,165].

Similarly, NUDT15 is critical in degrading the cytotoxic thioguanine triphosphate (TdGTP) into a less toxic form, thioguanine monophosphate. Variants in *NUDT15*, especially the p.R139C (*rs116855232*) mutation, found in the NUDT15*2 and NUDT15*3 haplotypes, are strongly associated with reduced enzymatic activity [167] and an increased risk of thiopurine-related toxicity, including severe myelosuppression [168]. Unlike *TPMT* polymorphisms, *NUDT15* variants are most prevalent in East Asian populations, with NUDT15 deficiency found in ~2% of East Asians compared to less than 1% in Europeans and Africans [168]. Heterozygous carriers of *NUDT15* variants may benefit from reduced thiopurine doses to mitigate toxicity, while homozygous individuals often require significant dose reductions or alternative therapies.

Guidelines from the Clinical Pharmacogenetics Implementation Consortium (CPIC) and the Dutch Pharmacogenetics Working Group (DPWG) recommend genotype-guided dosing for thiopurines, emphasizing dose reductions of up to 90% for patients homozygous for *TPMT* or *NUDT15* deficiencies and lower starting doses for heterozygous individuals [157,162,168]. Despite the utility of genetic testing, routine complete blood count (CBC) monitoring remains essential, as other factors, such as co-medications and non-genetic variability, can contribute to toxicity [155]. While pharmacogenetic testing is not mandatory before starting AZA therapy, it is strongly recommended by regulatory agencies and incorporated into pharmacogenetic guidelines to improve the safety and efficacy of treatment [155,160]. However, challenges such as population variability in allele frequencies and the incomplete understanding of rarer variants highlight the need for further research and integration of genetic testing into routine clinical practice [169].

### 4.6. Monoclonal and Polyclonal Antibodies

The widespread application of organ transplantation relies on general immunosuppressive drugs, but their long-term use is limited by chronic rejection and side effects, leading to plateaued transplant survival in recent years [170]. Organ rejection often necessitates secondary transplants, worsening the organ shortage and increasing morbidity and economic costs [171].

To overcome these challenges, innovative strategies are needed to reduce dependence on immunosuppressive drugs. International collaborations, such as EU-funded programs and the Immune Tolerance Network, aim to develop alternative solutions. Two key approaches are under investigation: a deletional strategy through donor bone marrow chimerism to reduce donor-reactive immune cells and an immune regulation strategy leveraging regulatory cells or pathways [172]. While chimerism protocols face challenges like conditioning regimen toxicity and graft-versus-host disease, immune regulation therapies have recently advanced to clinical testing, offering promising, targeted alternatives [172].

Regulatory cell therapy has gained recognition as a promising therapeutic approach for establishing immune regulation aimed at safeguarding organ allografts [173,174,175]. The core concept of this strategy involves the ex vivo expansion of specific regulatory immune cell populations, formulated as Cell-Based Medicinal Products (CBMPs), which are subsequently infused into transplant recipients [176].

CBMPs offer a cutting-edge strategy to mitigate the need for general immunosuppression in organ transplantation. While their safety has been demonstrated in living-donor kidney transplant recipients, reducing complications, they have not shown a significant decrease in rejection rates when combined with other immunosuppressive (IS) drugs during the first year post-transplantation [176].

Biologic agents, including antibodies, have been developed for use in induction therapy or for the treatment of transplant rejection. These agents modulate the immune response through various mechanisms. Induction therapies can target lymphocytes to inhibit their activation and proliferation, such as with the use of IL-2 receptor antagonists (IL-2RAs) [177].

Monoclonal antibodies (mAbs), produced from identical immune cells derived from a single parent cell, exhibit monovalent affinity, binding to a specific epitope.

Basiliximab is a chimeric monoclonal antibody that specifically targets the alpha chain of the interleukin-2 receptor (IL-2R). By binding to this chain, it inhibits the activation of the IL-2 receptor. Alemtuzumab facilitates the destruction of T and B lymphocytes, monocytes, and NK cells in peripheral blood, leading to a deep and sustained depletion of T lymphocytes, while B lymphocytes and monocytes experience a more temporary reduction [178]. Both agents exhibit high-affinity binding to the 55 kD alpha chain of the interleukin-2 receptor (IL-2R or CD25), thereby preventing the formation of the IL-2 binding site [179]. The IL-2R alpha chain plays a crucial role in the heterotrimerization of the IL-2 receptor complex, enhancing IL-2 binding affinity and driving the rapid clonal expansion of activated T lymphocytes. By specifically inhibiting T-cell activation, antibodies targeting the IL-2R alpha chain can disrupt the signaling cascade responsible for cellular proliferation, cytokine release, and subsequent tissue inflammation and acute rejection [180].

In contrast, polyclonal antibodies, derived from multiple cell lineages, target multiple epitopes [181]. Among these are polyclonal anti-thymocyte globulins (ATG), which are produced by immunizing animals with human lymphoid cells. The antibodies with the greatest efficacy target markers such as CD2, CD3, CD4, CD8, CD11a, CD18, CD25, CD28, CD40, and CD54, displaying a wider immunosuppressive activity than monoclonal antibodies [178]. However, unresolved issues remain regarding dose individualization, the therapeutic significance of their non-depletive effects, and the prediction of long-term outcomes [182].

Unlike traditional IS drugs, these agents, including ATG, are not metabolized by the hepatic cytochrome P450 system, which eliminates the possibility of tailoring their dosage through pharmacogenomic strategies. This limitation extends to rituximab, another widely used immunosuppressive agent. Rituximab, similar to basiliximab and ATG, has been employed pre-transplantation to enhance immunosuppression by targeting lymphocyte activity [183].

The mAbs are categorized as chimeric (-ximab), with variable regions originating from mice, humans, or other species; humanized (-zumab), primarily human with small fragments derived from mice; and fully human (-humab), developed using humanized mouse technology [181].

Currently, the immunosuppressive agents used in transplantation therapies highlight both the advancements made and the limitations of current clinical practices. While these drugs, including monoclonal antibodies, are essential tools in preventing organ rejection, their limited adaptability to pharmacogenomic strategies underscores the need for further progress toward more personalized medicine. Despite their potential, research studies and clinical trials specifically focusing on kidney transplantation are still limited, making it challenging to accurately assess the efficacy of monoclonal antibodies and their impact on long-term transplant and patient survival. Additionally, there is no universal consensus regarding the optimal dosing and timing of the administration of these agents. In most cases, the therapeutic decision must strike a delicate balance between expected benefits, the risks of long-term complications, and the economic costs of therapy. This scenario emphasizes the importance of ongoing research and a more targeted, personalized approach in the care of transplant patients.

### 4.7. NGS and Biotechnological Innovation for Personalized Medicine in Transplant

Although pharmacogenetic testing has been considered cost-effective, some studies present contrasting views, evaluating the approach as cost-saving. The implementation of pharmacogenetic NGS-derived tests surely requires a substantial initial investment, and costs regarding bioinformatic structure, clinical data experts for interpretation, and computational tools will need to be sustained throughout. Also, the nature of the tests will have a variable impact on the total costs [184]. Currently, commercial and standardized Next Generation Sequencing (NGS) panels for pharmacogenetic analysis in renal transplant recipients are not yet available. However, they are anticipated to play a crucial role in the future of transplant management, enabling precision and personalized therapy to enhance graft survival rates. In agreement, an NGS analysis has been recently proposed to validate the efficiency of an NGS analysis, based on 17 genes, in predicting acute rejection events in kidney-transplanted patients [185]. The results of this prospective observational study demonstrated that the NGS evaluation can be considered a valid non-invasive tool to evaluate kidney graft rejection.

Whilst having a strong potential in the management of kidney transplant patients, some ethical concerns come along with the use of pharmacogenetic testing. One of these is the risk of intrinsically providing the patient with ancillary information after having analyzed their genes for specific mutations. In other words, the pharmacogenetic testing performed on a patient’s DNA to discover how they would respond to a specific drug might also uncover clinically relevant information such as the increased risk to develop a certain disease. This might also impact close relatives, both in the vertical and horizontal lines, who may hold the same genotype [186]. At this time, a very few studies were performed on the correlation between any of the polymorphisms mentioned in this paper and the increased risk of developing a specific disease.

In conclusion, organ transplantation is one of the most effective life-saving procedures, involving the surgical replacement of a severely damaged or failing organ with one from a healthy donor. However, significant challenges remain, including long-term complications of immunosuppression and a critical shortage of donors. Over the years, research has focused on biotechnological innovations to overcome these obstacles. One promising approach is xenotransplantation, which involves transplanting organs from animals—such as genetically modified pigs—to humans. Genetic editing, particularly through CRISPR-Cas9, is essential to reduce adverse immune reactions and improve compatibility. Another avenue of research involves the creation of artificial organs or in vitro tissue engineering. Techniques like 3D bioprinting enable the layer-by-layer deposition of biological materials to construct functional tissues, while organoids, derived from pluripotent stem cells (PSC) cultivated on biological matrices, provide miniature in vitro models of organs. These advancements hold great potential for transplantation and regenerative medicine [187].

Beyond organ replacement, scientists are exploring ways to repair damaged organs using stem-cell-based therapies. Stem cells can be directly engrafted into the affected area or leveraged for their paracrine effects, as they secrete regenerative molecules such as growth factors, cytokines, and extracellular vesicles. While promising, further research is needed to translate these findings into clinical applications and eventually solve the hurdles associated with organ transplantation [188].

## 5. Discussion

Kidney transplantation has become a routine procedure in many transplant centers worldwide. The introduction of CsA and TAC as immunosuppressive therapies in transplanted patients significantly improved graft survival and quality of life [189]. Moreover, the advent of new drugs that do not interact with the calcineurin pathway, such as MPA, EVR, and SRL, has further enhanced clinical outcomes.

However, immunosuppression is influenced by significant PK and PD variability. Among the variables impacting PK and PD, the genetic characteristics of both the recipient and donor play a crucial role, particularly concerning genes involved in the metabolism and transport of administered drugs. Polymorphisms in genes related to the metabolism and transport of immunosuppressive drugs, which significantly modify the PK and PD of immunosuppressive drugs, have been identified and extensively studied, emphasizing the importance of considering genetic profiles when determining treatment regimens.

Genetic testing for individual patients, particularly in cases of treatment failure or adverse side effects, provides a comprehensive understanding of the patient’s response. This approach aids in tailoring immunosuppressive therapies, allowing for dose adjustments or the substitution of ineffective or toxic drugs with more suitable alternatives. Evidence suggests, in fact, that survival rates for both kidney allografts and transplant recipients improve when optimal immunosuppressive drug concentrations are achieved. To attain this, it is crucial to account for all factors influencing therapeutic regimens, including genetic factors.

There is ongoing debate about the routine implementation of pharmacogenetic analyses in kidney transplant recipients due to resource constraints in healthcare systems. A review of the literature on the economic viability of pharmacogenetic testing in renal transplantation highlights that testing prior to treatment initiation is the most promising approach. Incorporating such tests into routine clinical practice would become more feasible if the comprehensive costs of renal transplantation are fully evaluated. This underscores the need for future research examining the cost-effectiveness of these assays [129].

## 6. Conclusions

**Figure 5:**
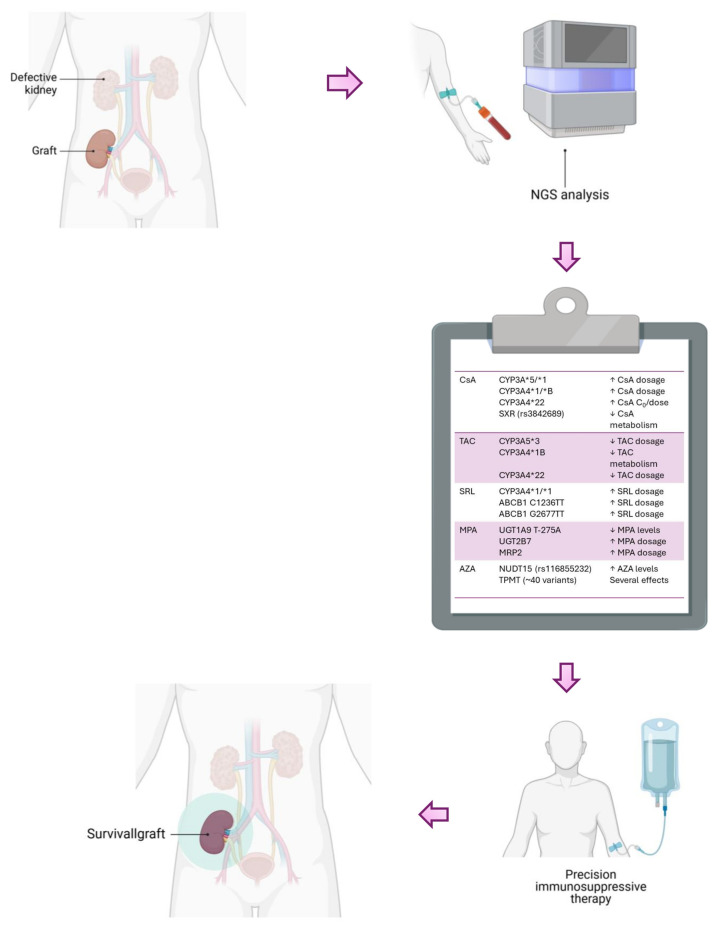
Pharmacogenetic analysis for precision therapy in kidney transplants. CsA: cyclosporine; TAC: Tacrolimus; SRL: Sirolimus; MPA: Mycophenolic Acid; AZA: Azathioprine. Created with BioRender.com (accessed on 14 January 2025).

In conclusion, identifying key polymorphisms associated with reduced efficacy in immunosuppressive therapy could prove invaluable (Figure 5). Incorporating genetic profiling into clinical practice may facilitate more precise immunosuppressive management, reducing adverse effects and improving patient outcomes. Future research should prioritize the integration of pharmacogenetic testing into routine practice, alongside cost-effectiveness evaluations, to support the widespread adoption of personalized medicine in kidney transplantation.
